# Low-protein diet does not alter reproductive, biochemical, and hematological parameters in pregnant Wistar rats

**DOI:** 10.1590/1414-431X20186602

**Published:** 2018-05-21

**Authors:** M.A.V. Barros, E.B. Andrade, R.G.N. Barros, I.K.M. Costa, I.C.L. Costa, G.F.A. Vitorino, J.J.C. Andrade, K.M. Paulino-Silva, V.O. Nogueira, J.L. de Brito Alves, J.H. Costa-Silva

**Affiliations:** 1 Laboratório de Nutrição, Atividade Física e Plasticidade Fenotípica Núcleo de Educação Física e Ciências do Esporte, Universidade Federal de Pernambuco, Vitória de Santo Antão, PE, Brasil; 2 Departamento de Nutrição, Centro de Ciências da Saúde, Universidade Federal de Paraíba, UFPB, João Pessoa, PB, Brasil

**Keywords:** Development, Epigenetics, Fertility, Gestation, Intrauterine growth, Nutrition

## Abstract

The aim of this study was to investigate the reproductive, biochemical, and hematological outcomes of pregnant rats exposed to protein restriction. Wistar rat dams were fed a control normal-protein (NP, 17% protein, n=8) or a low-protein (LP, 8% protein, n=14) diet from the 1st to the 20th day of pregnancy. On the 20th day, the clinical signs of toxicity were evaluated. The pregnant rats were then anesthetized and blood samples were collected for biochemical-hematological analyses, and laparotomy was performed to evaluate reproductive parameters. No sign of toxicity, or differences (P>0.05) in body weight gain and biochemical parameters (urea, creatinine, albumin, globulin, and total protein) between NP and LP pregnant dams were observed. Similarly, hematological data, including red blood cell count, white blood cell count, hemoglobin, hematocrit, red blood cell distribution width (coefficient of variation), mean corpuscular volume, mean corpuscular hemoglobin, mean corpuscular hemoglobin concentration, % lymphocytes, absolute lymphocyte count, platelet count, and mean platelet volume were similar (P>0.05) at the end of pregnancy. Reproductive parameters (the dam-offspring relationship, ovary mass, placenta mass, number of corpora lutea, implantation index, resorption index, and the pre- and post-implantation loss rates) were also not different (P>0.05) between NP and LP pregnant dams. The present data showed that a protein-restricted diet during pregnancy did not alter reproductive, biochemical, and hematological parameters and seems not to have any toxic effect on pregnant Wistar rats.

## Introduction

Gestation and lactation (perinatal period) are characterized by an intense process of hypertrophy, hyperplasia, and cellular differentiation (1). In this period, nutritional supplies are important for adequate intra-uterine growth and development of pups. Epidemiological and experimental reports have demonstrated that nutritional insults, such as the consumption of a low-protein diet during gestation and lactation, produce maternal and offspring metabolic dysfunction (2,3).

Pups from protein-restricted mothers, in the short-term, are able to adapt to a harmful environment to ensure their survival. Though this adaptation is beneficial in the short-term, offspring exposed to maternal malnutrition exhibit several long-term consequences, such as a higher predisposition to the development of non-communicable diseases (4).

In rats, for example, offspring exposed to protein-restriction during pregnancy and lactation exhibit augmentation of arterial blood pressure (5,6), insulin resistance (7), and higher levels of adipose tissue in adult life (8). It is well established that maternal diet induced-hypertension is related to mechanisms that include reduced nephron morphology and function, reduced glomerular filtration rate, dysfunction on the rennin angiotensin-aldosterone system (9), as well as sympathetic-respiratory dysfunctions (10). Besides that, changes in muscle glucose metabolism by expression decrease in protein kinase C (11) and decrease in hexokinase activity (12) are related with insulin resistance and increased susceptibility to diabetes in malnutrition animals.

The phenomenon that links events experienced *in utero* with predisposition to diseases in adulthood is denominated “phenotypic plasticity”, and refers to the ability of an organism to react to an internal and external environmental input with a change in the form, state, movement or rate of activity without genetic changes (13,14).

Although there are a number of studies showing the relationship between maternal malnutrition and non-communicable disease in adult offspring, none has specifically addressed the effects of a protein-restricted diet on mothers and maternal-fetal coupling. Previous studies have demonstrated that protein-restricted diets during gestation produce important morphological and functional dysregulation at placental levels (15,16). In addition, protein-restricted pregnant dams exhibit decreased secretion of insulin (17).

It is known that dietary content is often an important environmental determinant of the toxicological activity. Thus, change in maternal and offspring body weight are viewed collectively as indicators of maternal and developmental toxicity, respectively (18). Besides that, clinical observations are an important approach for the identification of maternal toxicity and alterations in general homeostasis (18,19).

Despite these findings, the reproductive, biochemical, and hematological parameters in pregnant rats exposed to protein restriction remain to be clarified. Therefore, the present study aimed to assess the effects of maternal protein restriction on the reproductive, biochemical, and hematological status of pregnant rats.

## Material and Methods

### Animals

Rats of the Wistar lineage, obtained from the Academic Center of Vitoria (Federal University of Pernambuco, Brazil) and weighing 210–250 g, were used and kept under standard environmental conditions (25±2°C; 12:12 h dark/light cycle). Water and chow diet were available *ad libitum.* The experimental protocol was approved by the Animal Experimentation Ethics Committee of the Centro de Ciências Biológicas, Universidade Federal de Pernambuco (Process No. 23076.016525/2014-92).

### Diets

Both the normal-protein (17% of protein) and low-protein (8% of protein) diets were prepared at the Laboratório de Nutrição Experimental-CAV, Universidade Federal de Pernambuco, according to the American Institute of Nutrition (AIN-97). The diets were isoenergetic and were offered during pregnancy. Only the amounts of protein and carbohydrate were changed in the diets (Table 1) (20).

**Table 1.** Nutritional composition of the experimental diets.

Nutrient	Normal protein (17% protein)	Low protein (8% protein)
Casein (85% purity)	20.0	9.41
Dextrin cornstarch	13.0	13.2
Cellulose	5.0	5.0
Sucrose	10.0	10.0
Cornstarch	39.74	50.34
Soybean oil	7.0	7.0
Choline	0.25	0.25
Methionine	0.3	0.3
Vitamin mix	1.0	1.0
Mineral mix	3.5	3.5
Energy density (kJ/g)	16.26	16.26

Data are reported as g/100 g diet.

### Experimental protocol

The rats were first mated (2 females for 1 male). The day on which spermatozoa were identified in a vaginal smear was considered the date of conception (day 1 of pregnancy), and pregnant rats were transferred to individual cages. Two experimental groups were designated according to diet manipulation: mothers fed a 17% protein diet (normal-protein group, NP, n=8) and mothers fed an 8% casein diet (low-protein group, LP, n=14). Water was available *ad libitum* from the 1st to the 20th day of pregnancy (21,22). During pregnancy, body weight and food and water intake were recorded weekly.

On the 20th day of pregnancy, the clinical signs of toxicity (piloerection, diarrhea, salivation, alteration in locomotor activity, changes in behavior or signs of vaginal bleeding) were evaluated. Posteriorly, the rats were anesthetized with ketamine (80 mg/kg) and xylazine (10 mg/kg) and blood samples (about 1–2 mL) were collected by plexus retro-orbital disruption, using capillary tubes for hematological and biochemical studies, with and without anticoagulant, respectively (23). The animals were then laparotomized and their uterine horns removed to determine reproductive parameters (21).

### Biochemical and hematological analysis

Hematological analysis was performed using an automatic hematological analyzer (KX-21N, Sysmex, Japan). The parameters included: red blood cell count, white blood cell count, hemoglobin, hematocrit, red blood cell distribution width coefficient of variation, mean corpuscular volume, mean corpuscular hemoglobin, mean corpuscular hemoglobin concentration, % lymphocytes, absolute lymphocyte count, platelet count, and mean platelet volume (23). For biochemical analysis, the blood was centrifuged at 1480 *g* for 10 min at room temperature to obtain serum, which was stored at -20°C until determination of the following parameters: total protein, albumin, globulins, blood urea nitrogen, and creatinine. The dosages were chosen using Cobas Mira (Roche, USA) automation with Boehringer Ingelheim¯ (USA) biochemical kits.

### Reproductive parameters

On the 20th day of pregnancy, the rats were laparotomized and their uterine horns removed. The number of implants, resorptions, and the number of live and dead fetuses was then recorded. The fetuses and placentae were observed for macroscopic abnormality. The ovaries were weighed and the corpora lutea were counted. From these data, the implantation index (total number of implantation sites/total number of corpora lutea ×100), the resorption index (total number of resorption sites/total number of implantation sites ×100), the pre-implantations (number of corpora lutea - number of implantations/number of corpora lutea ×100) and the post-implantation loss rate (number of implantations - number of live fetuses/number of implantations ×100) were calculated.

### Statistical analysis

Student's unpaired *t*-test was used to evaluate significant differences between the normal- and low-protein groups. One-way ANOVA followed by the Newman-Keuls tests were used to evaluate significant differences in hematological parameters. The pre-implantation and post-implantation loss rates and the implantation and resorption indexes were analyzed using Kruskal-Wallis and chi-square tests, respectively. The significance level was set at P<0.05.

## Results

Low-protein diet consumption from the 1st to 20th day of pregnancy did not produce any death or clinical signs of toxicity in the pregnant rats. Maternal food consumption was affected in the 1st week of pregnancy, but no differences were noted in the following weeks. However, malnourished dams had a smaller protein intake than control dams in all weeks analyzed. A reduction in water intake in the 2nd week of pregnancy (Table 2) was also observed.

**Table 2.** Consumption of female rats submitted to a normal- (NP group, 17% protein) or low-protein diet (LP group, 8% protein) during pregnancy.

Period of pregnancy	Food Intake (g)		Protein Intake (g)		Water Intake (mL)	
	NP	LP	NP	LP	NP	LP
Week 1	111±14	146±9*	18±1	12±1*	166±14	149±8
Week 2	123±7	130±4	21±1	10±1*	177±7	151±7*
Week 3	93±9	89±4	15±1	6±1*	109±9	115±6

Data are reported as means±SE. *P<0.05 compared with NP group (unpaired Student's *t*-test).

The hematological profiles of NP and LP pregnant rats are presented in Table 3. NP and LP dams exhibited similar (P>0.05) hematological parameters in pre- and late pregnancy.

**Table 3.** Hematological parameters of female rats subjected to a normal- (NP group, 17% protein) or low-protein diet (LP group, 8% protein) during pregnancy.

Item (unit)	Baseline (before mating)		Pregnancy	
	NP	LP	NP	LP
WBC (×10^3^/µL)	14.3±1.8	16.7±1.4	9.2±2.1	11.4±1.9
RBC (×10^6^/µL)	7.6±0.3	7.6±0.1	7.6±0.2	7.6±0.3
Hemoglobin (g/dL)	13.9±0.2	13.9±0.2	14.4±0.6	14.3±0.5
Hematocrit (%)	46.3±0.6	45.9±0.6	43.2±1.1	43.6±0.9
RDW CV (%)	11.9±0.2	13.1±0.4*	14.6±0.8	13.9±0.9
MCV (fL)	60.8±0.5	60.1±0.6	56.7±0.7	57.5±0.9
MCH (pg)	18.3±0.2	18.2±0.3	18.9±0.2	18.9±0.2
MCHC (g/dL)	30.1±0.1	30.2±0.2	33.4±0.6	32.8±0.6
LYM (%)	70.9±1.1	75.1±1.8	71.8±2.6	73.1±2.9
LYM ABS (×10^3^/µL)	10.1±1.2	12.5±1.1	11.6±0.7	10.9±1.3
Platelets (×10^3^/µL)	513.1±43.6	655.2±48.8*	553.5±48.3	640.9±55.5
MPV (fL)	7.1±0.1	6.8±0.1*	6.9±0.2	6.8±0.2

Data are reported as means±SE. WBC: white blood cells; RBC: red blood cells; RDW CV: red blood cell distribution width coefficient of variation; MCV: mean corpuscular volume; MCH: mean corpuscular hemoglobin; MCHC: mean corpuscular hemoglobin concentration; LYM: lymphocytes; LYM ABS: lymphocytes absolute value; MPV: mean platelet volume. *P<0.05 compared with NP group (unpaired Student's *t*-test).

**Figure 1.**
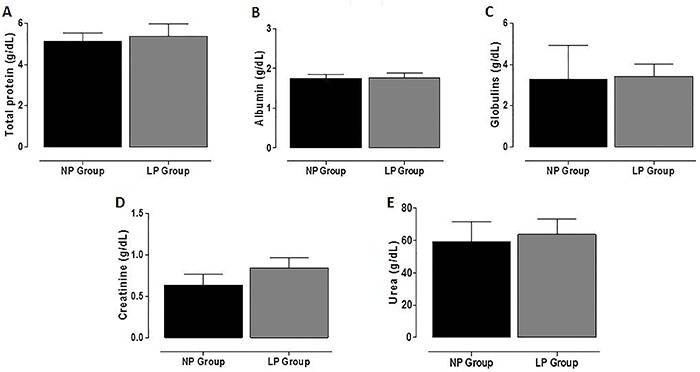
Serum biochemical parameters. Total protein (*panel A*), albumin (*panel B*), globulins (*panel C*), creatinine (*panel D*), and urea (*panel E*) of female rats subjected to a normal- (NP group, 17% protein) or low-protein diet (LP group, 8% protein) during pregnancy. Data are reported as means±SE and the comparison between groups was done by unpaired Student's *t*-test (P>0.05).

Biochemical analysis revealed that protein-restriction during pregnancy did not alter albumin (NP=1.7±0.1 *vs* LP=1.8±0.1 g/dL; n=7; P>0.05), globulins (NP=3.3±0.5 *vs* LP=3.4±0.6 g/dL; n=7; P>0.05), and total protein (NP=5.1±0.4 *vs* LP=5.4±0.6 g/dL; n=7, P>0.05). Similarly, urea (NP=59±12 *vs* LP=64±9 g/dL; n=7, P>0.05) and serum creatinine (NP=0.6±0.1 *vs* LP: 0.8±0.1 g/dL; n=7, P>0.05) were similar between the NP and LP groups (Figure 1).

All pregnant females were found to have viable fetuses, observed after a caesarian section. No fetuses with external malformations were observed. In addition, there were no differences between NP and LP dams regarding the number of fetuses of each dam (offspring/dam relationship), the number of corpora lutea, and ovary weights. Likewise, maternal low protein intake did not cause any changes in the implantation and resorption indexes or the pre- and post-implantation loss rates (Table 4).

**Table 4.** Reproductive parameters of female rats subjected to a normal- (NP group, 17% protein) or low-protein diet (LP group, 8% protein) during pregnancy.

Reproductive parameters	NP group	LP group
Pregnant rats (n)	8	14
Mass gain in the pregnancy period (g)^a^	75±11	70±5
Mass gain in the organogenic period (g)^a^	35±4	24±3
Offspring/dam relationship^a^	12±1	11±1
Ovary mass (mg/100g)^a^	37.9±3.6	37.2±4.5
Fetus mass (g)^a^	2.5±0.1	2.6±0.2
Placentae mass (g)^a^	37±4	38±3
Number of corpora lutea^a^	13±1	12±1
Implantation index (%)^a^	94±2	93±2
Resorption index (%)^a^	2±1	4±2
Pre-implantation loss (%)^b^	9	6
Post-implantation loss (%)^b^	0	7

^a^Data are reported as means±SE and were analyzed by the unpaired Student's *t*-test (P>0.05). ^b^Data are reported as median percent and were analyzed by Kruskal-Wallis and chi-square tests (P>0.05).

## Discussion

The main finding of this study is that a low-protein diet did not produce any death or toxic clinical signs in pregnant rats, nor changed the biochemical, hematological, and reproductive parameters of the animals.

It is known that maternal parameters such as body weight gain, food consumption, and clinical signs of toxicity enable a clear evaluation of the integrity of maternal homeostasis (24). We observed that a protein-restricted pregnant rat had a significant reduction in protein intake during the entire pregnancy period (about 50%). Interestingly, the low protein intake did not decrease maternal weight gain, suggesting that the homeostatic mechanism is able to provide normal development during pregnancy.

Besides that, in the first week of pregnancy, the LP pregnant rats exhibited higher food consumption compared to pregnant rats fed on a normal diet during the same period. Similarly, in a study with different protein-calorie diets during lactation in rats, an increased diet intake during the beginning of lactation in the low-protein group was demonstrated (25). It is known that feeding is controlled by a central feeding system that is regulated by a balance between monoamines and neuropeptides. Thus, the animals possibly present a mechanism to compensate for a low-protein diet, due to a regulatory system involving gastrointestinal and metabolic aspects mediated by neural structures, which may have stabilized in the second week of pregnancy.

Despite the fact that the protein-restricted dams did not present differences in body weight, offspring from malnourished dams during pregnancy and/or lactation have been shown to have a lower weight and shorter length at birth as well as a higher incidence of mortality in the first days of life (6,26-29). Recent studies have shown that offspring exposed to perinatal protein restriction exhibit a number of dysfunctions in respiratory, cardiovascular, renal, behavioral, and reproductive levels during their lives. As the developing organism is capable of adapting to various environments, these physiological alterations over the course of life appear to be the result of complex gene-environment interactions, resulting from epigenetic changes during their critical developmental time window (30,31).

In the same manner, studies have also shown that maternal-fetal coupling suffers injury under maternal malnutrition, with the placenta being the focus of these studies. For example, a low-protein diet during pregnancy induced placental oxidative stress (32) as well as mitochondrial alterations and degenerative processes, suggesting a premature aging of the placenta (33). Although previous studies found no change in the weight of the placenta (34), function of the placenta appears to be compromised by low-protein diet intake.

To understand this, we investigated if this diet could change the biochemical and hematological parameters as well as compromise the reproductive capacity of the mother. Our data provided new insights into the effects of protein-restriction during pregnancy, demonstrating that after exposure to a protein-restricted diet, NP and LP pregnant rats showed similar hematological profiles. Thus, all parameters remained within the reference range for the species. Prestes-Carneiro et al. (35) have reported that exposure to a low-protein diet from 12 days of lactation can induce alterations in red blood cell count in the offspring, which is never restored completely even after a normal-protein diet is supplied. Our study shows clearly that protein restriction during pregnancy does not modify maternal hemostasis.

On the other hand, our data also showed that a low-protein diet during pregnancy did not change maternal serum levels of the albumin, total protein, globulin, urea, and creatinine. We hypothesized that physiological synthesis of the protein and its metabolism during pregnancy is not affected by lower protein and amino acid intakes.

Regarding reproductive parameters, we also found similar ovarian mass and the number of corpora lutea in both the protein restriction group and control group. These findings indicated normal development of corpora lutea and suggested that the production of progesterone is not influenced by low-protein diet (36).

Implantation index and pre-implantation loss rate evaluate blastocyst implantation in the uterus. These parameters were similar in both control and malnourished groups, suggesting normal reproductive capacity (36). The resorption index and post-implantation loss rate establish correlations between the number of implanted blastocysts and those that do not develop (24). When the implanted blastocysts do not develop, they are known as “resorptions”, which indicate failure in the development of the embryo. In this study, there was no statistical difference between control and malnourished groups for the resorption index and post-implantation loss rate, indicating normal development of the implanted blastocysts.

Although our results showed no damage in low-protein dams, it is known that a restriction of protein during pregnancy may induce changes in other pathways, such as glucoses metabolism and insulin secretion (37,38). A decrease in (Ca^2+^)i, as well as changes in gene expression in pancreatic islets (39) can explain the decreased insulin secretion in malnourished animals. A study also demonstrated that physical training before and during pregnancy attenuated the effects of a low-protein diet on the secretion of insulin (17).

In conclusion, the present study showed that a low-protein diet during pregnancy did not change the hematological, biochemical, and reproductive parameters, and seems not to have any toxic effect on pregnant Wistar rats. These data strengthen the plasticity phenotype theory, in which the adaptive mechanisms elicited by the maternal organism are responsible for providing normal nutrient contents to the fetus during exposure to maternal protein restriction. However, other parameters indicate alterations in maternal-fetal coupling induced by protein restriction in the literature and therefore, the long-term effects of this maternal physiological adaptation on the offspring needs to be further studied.
